# Supplementary material for: Redox-triggered switching in three-dimensional covalent organic frameworks
Source: Nat Commun. 2020 Oct 1;11:4919. doi: 10.1038/s41467-020-18588-1 (PMC7531008; doi:10.1038/s41467-020-18588-1)
Supplement: Supplementary file 1 — Supplementary Information [file 41467_2020_18588_MOESM1_ESM.docx]

**Supplementary Information**

**Redox-triggered Switching in Three-Dimensional Covalent Organic Frameworks – Gao *et al*.**

**This file includes:**

Supplementary Methods 1 to 8

Supplementary Figure 1 to 32

Supplementary Table 1 to 5

Supplementary References

Supplementary Method 1

All reagents and solvents were purchased from commercial sources and used without further purification. Mesitylene, 1,2-dichlorobenzene and acetonitrile were purchased from Acros. Tetrabromohydroquinone, *p*-benzoquinone and ascorbic acid were purchased from Innochem or Adamas. TAPM was synthesized according to the previously reported literature.^1^

^1^H and ^13^C NMR spectra were measured on a Bruker Fourier 400 M spectrometer. High resolution mass spectra were collected on Bruker Solarix. Elemental analysis was conducted on a Flash EA 1112. Fourier transform infrared (FT-IR) spectra were recorded on a Nicolet iN10 micro FTIR Spectrometer. Powder X-ray diffraction (PXRD) patterns were obtained on a Rigaku Smartlab X-Ray diffractometer with Cu Kα line focused radiation at 40 kV and 50 mA. Thermogravimetric analysis (TGA) from 30-800 °C was carried out on a TGA-Q500 in nitrogen atmosphere using a 10 °C min^-1^ ramp without equilibration delay. Field-emission scanning (FE-SEM) was performed on a Zeiss ∑IGMA operating at an accelerating voltage ranging from 0.1 to 20 kV. The sample was prepared by dispersing the material onto wafer or conductive adhesive tapes attached to a flat aluminum sample holder and then coated with gold. Molecular modeling was carried out using Materials Studio suite of programs (Accelrys Inc.). High resolution solid-state NMR spectra were conducted at ambient pressure on a Bruker AVANCE III 400M spectrometer. The nitrogen isotherms were measured at 77 K using an Autosorb-iQ (Quantachrome) surface area size analyzer. Before measurement, the samples were degassed in vacuum at 120 °C for 12 h. Oil-free vacuum pumps and oil-free pressure regulators were used for measurements to prevent contamination of the samples during the degassing process and isotherm measurement.

**COFs hydrolysis:** To figure out the oxidation or reduction degree, we hydrolyzed the COFs powders at different reaction times. The hydrolysis process is listed as follow: A Pyrex tube was charged with isolated COFs powders (10 mg), acetonitrile (2 mL) and 3M HCl (0.1 mL). After being degassed by freeze-pump-thaw technique for three times and then sealed under nitrogen, the tube was stirred overnight at 80 °C. After cooling to room temperature, the solvent was removed under reduced pressure. Then the residues were collected for ^1^H NMR study.

Supplementary Method 2

Chemical Synthesis of Precursors

**Supplementary Figure 1∣**Synthesis of TPB-HQ

**Synthesis of Compound 1:** A solution of tetrabromohydroquinone (1.7 g, 4.0 mmol) in THF (8 mL) was added dropwise to a stirred suspension of NaH (0.4 g, 60% dispersion in mineral oil, 10 mmol) in THF (5 mL) at 0 °C under argon. After stirring for 5 min, MOMCl (0.9 mL, 12 mmol) was added slowly and the reaction mixture was stirred for an additional 2 h at 0 °C. Then, the reaction contents were quenched by addition of water and extracted with EtOAc (3 × 30 mL). The combined organic layers were then washed with 1 M NaOH and brine, dried over anhydrous Na_2_SO_4_. After that, the solvent was evaporated under reduced pressure, and the crude product was purified by column chromatography [SiO_2_ : CH_2_Cl_2_] to yield compound **1** as a white solid (1.6 g, 78% yield). ^1^H NMR (400 MHz, CDCl_3_, ppm): *δ* = 5.15 (s, 4H), 3.72 (s, 6H). ^13^C NMR (100 MHz, CDCl_3_, ppm): *δ* = 150.4, 121.8, 100.0, 58.8.

**Synthesis of Compound 3:** It was synthesized according to the literature^2^. A mixture of **2** (20.0 g, 74.0 mmol), bis(pinacolato)diboron (18.8 g, 74.0 mmol), Pd(dppf)Cl_2_·CH_2_Cl_2_ (3.0 g, 3.7 mmol), and potassium acetate (29.1 g, 296.5 mmol) in dioxane (300 mL) was refluxed overnight under nitrogen. After cooling to room temperature, the solids were filtered off and washed with ethyl acetate. The filtrate was evaporated under reduced pressure, and the crude product was purified by recrystallization three times from ethanol to yield compound **3** as a white solid (16 g, 68% yield).

**Synthesis of Compound 4:** Compound **1** (1.0 g, 2.0 mmol), compound **3** (3.1 g, 9.7 mmol), palladium tetrakis(triphenylphosphine) (0.23 g, 0.2 mmol), and potassium carbonate (2.2 g, 15.6 mmol) were added to a flask containing 1,4-dioxane/H_2_O (50 mL/5 mL). The mixture was heated under nitrogen at 90 °C for 3 days. After cooling to room temperature, the solvent was removed under reduced pressure. Then the residues were dissolved in CH_2_Cl_2_, washed with water and brine, dried over anhydrous Na_2_SO_4_. After that, the solvent was evaporated under reduced pressure, and the crude product was purified by column chromatography [SiO_2_: CH_2_Cl_2_/ethylacetate = 40/1] to yield compound **3** as a white solid. ^1^H NMR (400 MHz, CDCl_3_, ppm): *δ* = 7.30 (d, *J* = 8.1 Hz, 8H), 7.16 (d, *J* = 8.1 Hz, 8H), 5.28 (s, 4H), 4.02 (s, 4H), 3.73 (d, *J* = 11.0 Hz, 8H), 3.59 (d, *J* = 10.9 Hz, 8H), 2.32 (s, 6H), 1.26 (s, 12H), 0.78 (s, 12H).

**Synthesis of** **1,2,4,5-tetrakis-(4-formylphenyl)-3,​6-​dihydroxy-benzene (TPB-HQ):** Trifluoroacetic acid (75 mL) was added to a solution of compound **4** (3.0 g, 3.1 mmol) in CH_2_Cl_2_ (150 mL), and then the mixture was stirred overnight at room temperature. Subsequently, the reaction was quenched by adding potash solution. The mixture was filtered off and washed with water and methanol to give TPB-HQ as a white solid. ^1^H NMR (400 MHz, DMSO-*d*_6_, ppm): *δ* = 9.90 (s, 4H), 7.74 (s, 2H), 7.71 (d, *J* = 8.1 Hz, 8H), 7.34 (d, *J* = 8.0 Hz, 8H). ^13^C NMR (100 MHz, DMSO-*d*_6_, ppm): *δ* = 193.4, 144.7, 143.9, 134.8, 132.4, 130.7, 129.1. HR-MS (EI): m/z calcd for C_34_H_22_O_6_: 526.1416 [M]^+^; found: 526.1413 [M]^+^.

**Supplementary Figure 2∣**Synthesis of TPB-Q

**Synthesis of 2,3,5,6-Tetrakis(4-formylphenyl)-2,5-cyclohexadiene-1,4-dione (TPB-Q):** TPB-HQ (100 mg, 0.2 mmol) was added to a flask containing a solution of Fe(ClO_4_)_3_ in acetonitrile (20 mL, 30 mmol L^-1^), and the mixture was stirred for 1 h at room temperature. Subsequently, the solvent was removed in vacuum, and the crude product was purified by column chromatography [SiO_2_: CH_2_Cl_2_/ethylacetate = 20/1] to yield TPB-Q as an orange solid (89 mg, 89% Yield). ^1^H NMR (400 MHz, DMSO-*d*_6_, ppm): *δ* = 9.94 (s, 4H), 7.80 (d, *J* = 8.3 Hz, 8H), 7.40 (d, *J* = 8.1 Hz, 8H). ^13^C NMR (100 MHz, DMSO-*d*_6_, ppm): *δ* = 193.3, 185.5, 142.8, 139.4, 136.0, 131.7, 129.1. HR-MS (EI): m/z calcd for C_34_H_20_O_6_: 525.1333 [M+H]^+^; found: 525.1330 [M+H]^+^.

Supplementary Method 3

Synthesis of 3D-TPB-COF-HQ and 3D-TPB-COF-Q

**Supplementary Figure 3∣**Synthesis of 3D-TPB-COF-HQ and 3D-TPB-COF-Q

**Synthesis of 3D-TPB-COF-HQ:** A Pyrex tube was charged with TAPM (30.4 mg; 0.08 mmol), 1,2,4,5-tetrakis-(4-formylphenyl)-3,6-dihydroxy-benzene (TPB-HQ) (42.0 mg, 0.08 mmol), mesitylene (7.2 mL), *n*-BuOH (0.8 mL) and 12 M aqueous acetic acid (0.8 mL). After being degassed by freeze-pump-thaw technique for five times and then sealed under vacuum, the tube was placed in an oven at 120 °C for 7 d. The resulting precipitate was filtered, exhaustively washed by Soxhlet extractions with tetrahydrofuran, methanol and dichloromethane for 4 d, dried at 120 °C under vacuum for 12 h. The 3D-TPB-COF-HQ was isolated as pale yellow powder (52.9 mg, 79% yield). Elemental analysis for the calculated: C, 84.87%; H, 4.59%; N, 6.71%. Found: C, 80.11%; H, 4.58%; N, 6.62%.

**Synthesis of 3D-TPB-COF-Q:** A Pyrex tube was charged with 3D-TPB-COF-HQ (20.0 mg), *p*-benzoquinone (21.8 mg) and CH_3_CN (5.0 mL). The tube was sealed under air and stirred for 90 min at 60 °C. The resulting precipitate was filtered and exhaustively washed by CH_3_CN, dried at 120 °C under vacuum for 12 h. The 3D-TPB-COF-Q was isolated as orange powder (18 mg, 90 %). From the H NMR spectrum of digested 3D-TPB-COF-Q, the content of quinone is about 90% (see Supplementary Method 7 for details). It should be emphasized here, we have tried to directly synthesize 3D-TPB-COF-Q from TAPM and TPB-Q many times, but unfortunately failed.

**Further reduction of 3D-TPB-COF-Q:** 3D-TPB-COF-Q (20 mg) was added to a tube containing a solution of ascorbic acid in MeOH (5.0 mL, 0.2 mol L^-1^). After that, the mixture was stirred at 30 °C for 90 minutes. The resulting 3D-TPB-COF-HQ(R) (18.4 mg, 92 %) was filtered and exhaustively washed by MeOH and DCM, dried under vacuum. From the ^1^H NMR spectrum of digested 3D-TPB-COF-HQ(R), all of quinone units was reduced to hydroquinone groups (see Supplementary Method 7 for details).

Supplementary Method 4

**FT-IR Spectroscopy Analysis**

**
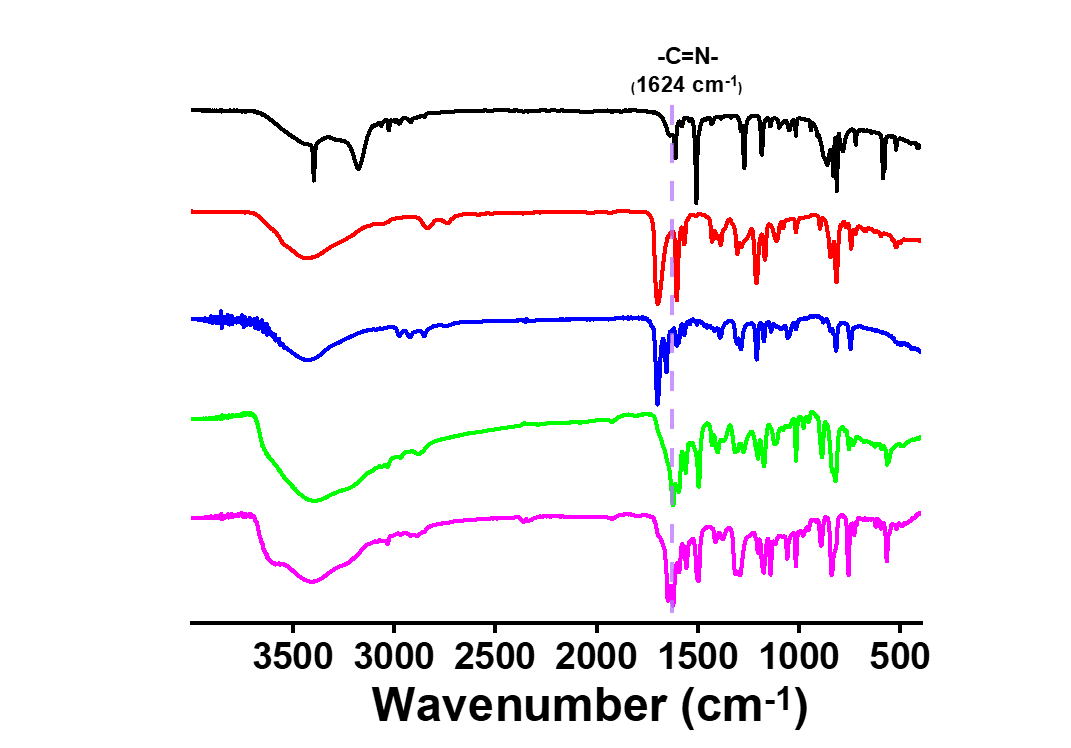
**

**Supplementary Figure 4∣**FT-IR spectra of TAPM (black curve), TPB-HQ (red curve), TPB-Q (blue curve), 3D-TPB-COF-HQ (green curve) and 3D-TPB-COF-Q (purple curve).

**^13^C Solid-State NMR Spectroscopy**

High resolution solid-state NMR spectra were conducted at ambient pressure on a Bruker AVANCE III 400M spectrometer using a standard CP-TOSS pulse sequence (cross polarization with total suppression of sidebands) probe with 4 mm (outside diameter) zirconia rotors. Cross-polarization with TOSS was used to acquire ^13^C data at 100.37 MHz. The ^13^C ninety-degree pulse widths were 4 µs. The decoupling frequency corresponded to 72 kHz. The TOSS sample-spinning rate was 12 kHz. Recycle delays was 3 s. For 3D-TPB-COF-HQ, *δ* = 160.1, 150.7, 144.6, 140.7, 135.3, 131.6, 114.7, 64.0. For 3D-TPB-COF-Q, *δ* = 186.4, 160.2, 148.7, 144.6, 137.0, 130.7, 116.5, 63.8.


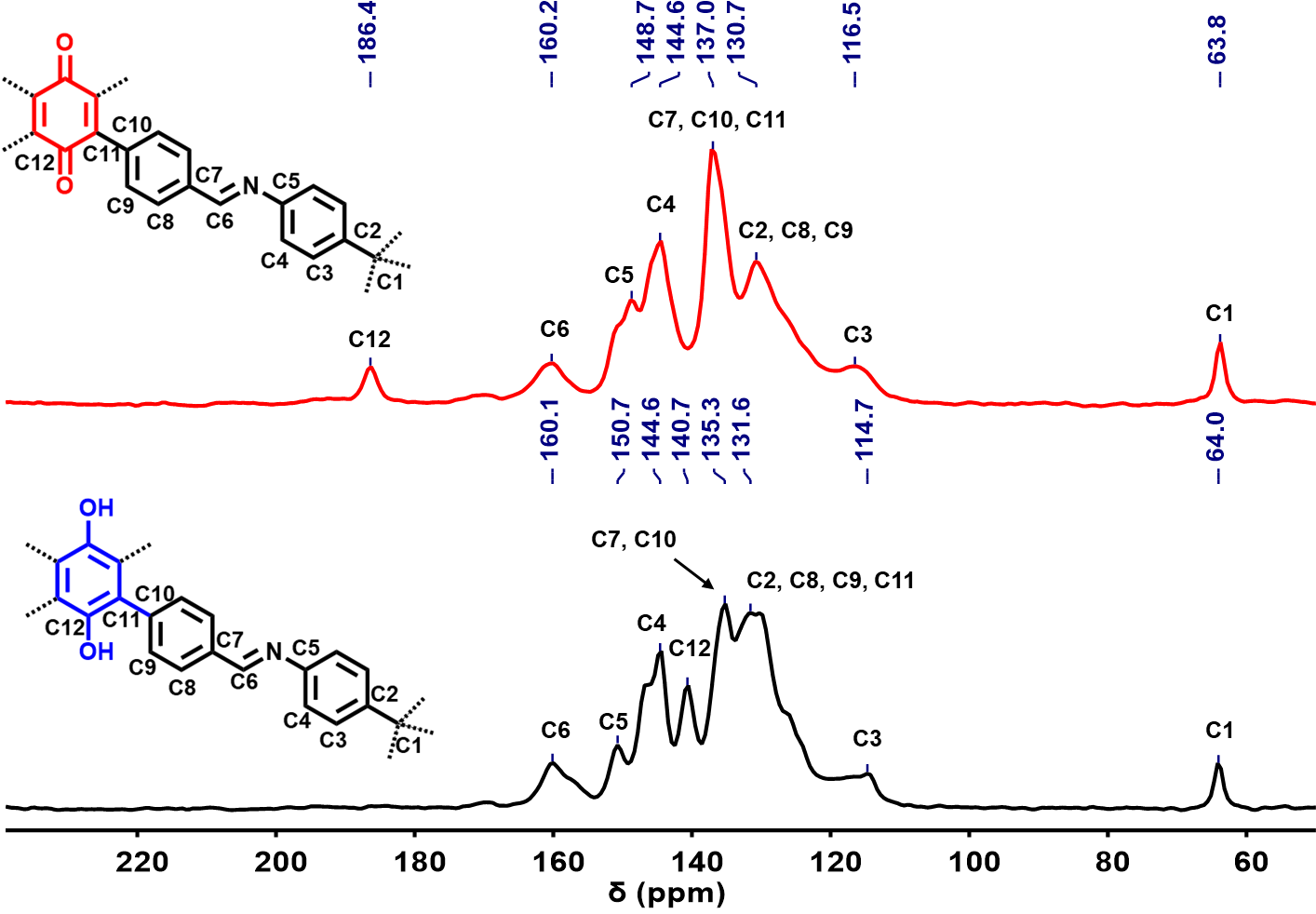


**Supplementary Figure 5∣**^13^C solid-state NMR spectrum of 3D-TPB-COF-HQ (black curve) and 3D-TPB-COF-Q (red curve).

**Thermogravimetric Analysis**


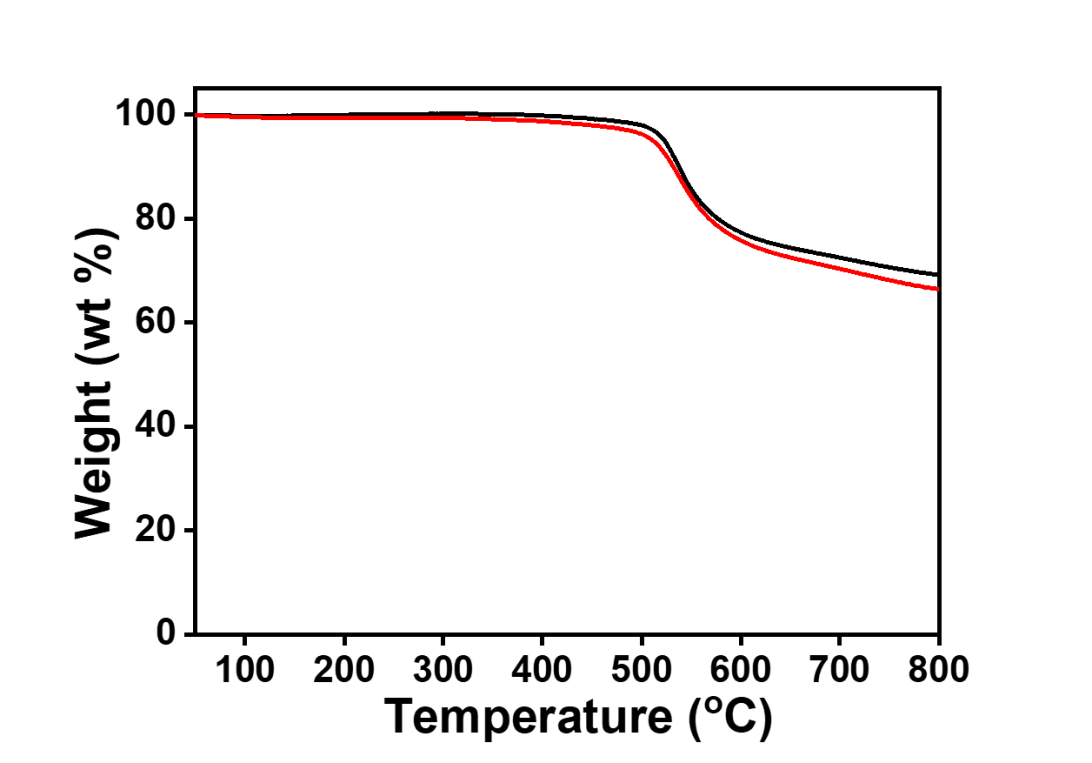


**Supplementary Figure 6∣**TGA profile of 3D-TPB-COF-HQ (black curve) and 3D-TPB-COF-Q (red curve).

**Scanning Electron Microscopy Images**

**Supplementary Figure 7∣**SEM images of 3D-TPB-COF-HQ (a, b), 3D-TPB-COF-Q (c, d) and 3D-TPB-COF-HQ(R) (e, f). Scale bar: 2 μm (a), 1 μm (b), 2 μm (c), 2 μm (d), 2 μm (e), 2 μm (f).

**Nitrogen Sorption Isotherm Measurements**

Before measurement, the samples were degassed in vacuum at 120 °C for 12 h. A liquid N_2_ bath was used for adsorption measurements at 77 K. To provide high accuracy and precision in determining *P*/*P*_0_, the saturation pressure *P*_0_ was measured throughout the N_2_ analyses by means of a dedicated saturation pressure transducer, which allowed us to monitor the vapor pressure for each data point. The Brunauer-Emmett-Teller (BET) method was utilized to calculate the specific surface areas. To estimate pore size distributions, N_2_ isotherms were analyzed by using nonlocal density functional theory (NLDFT) for N_2_ adsorption at 77 K based on a carbon model containing slit pores.


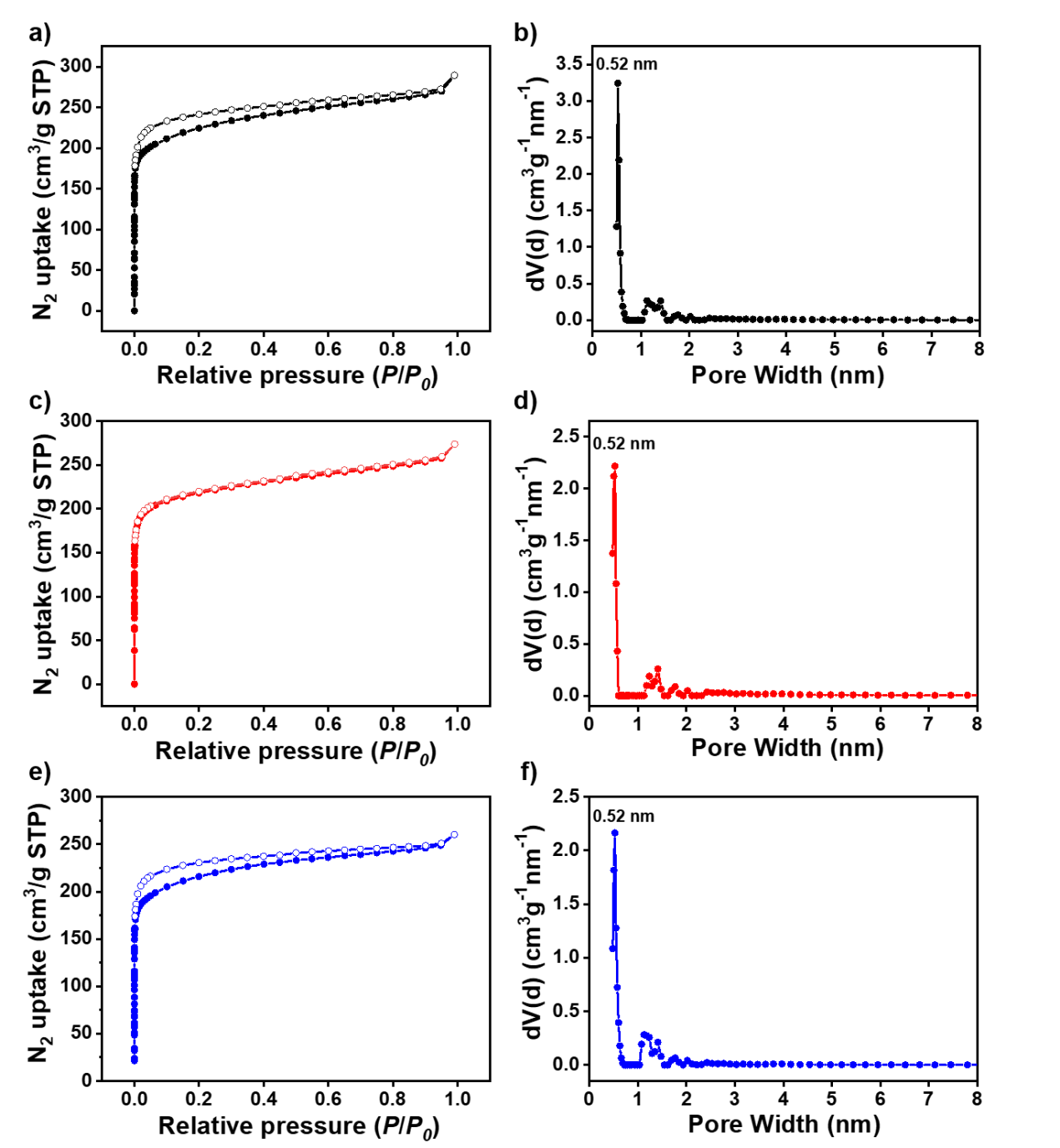


**Supplementary Figure** **8∣**N_2_ adsorption−desorption isotherms of 3D-TPB-COF-HQ (a), 3D-TPB-COF-Q (c), and 3D-TPB-COF-HQ(R) (e) at 77 K. Pore size distributions of 3D-TPB-COF-HQ (b), 3D-TPB-COF-Q (d), and 3D-TPB-COF-HQ(R) (f).

**Stability in Different Solvents**

In a typical experiment, 10 mg of 3D-TPB-COF-HQ or 3D-TPB-COF-Q was immersed in common solvents (DMF, DMSO, CH_3_CN, MeOH, H_2_O, 0.1M HCl) for 24 hours to test the stability of both 3D COFs. After that, the powder was filtrated, washed with tetrahydrofuran and dichloromethane and dried to measure PXRD patterns.


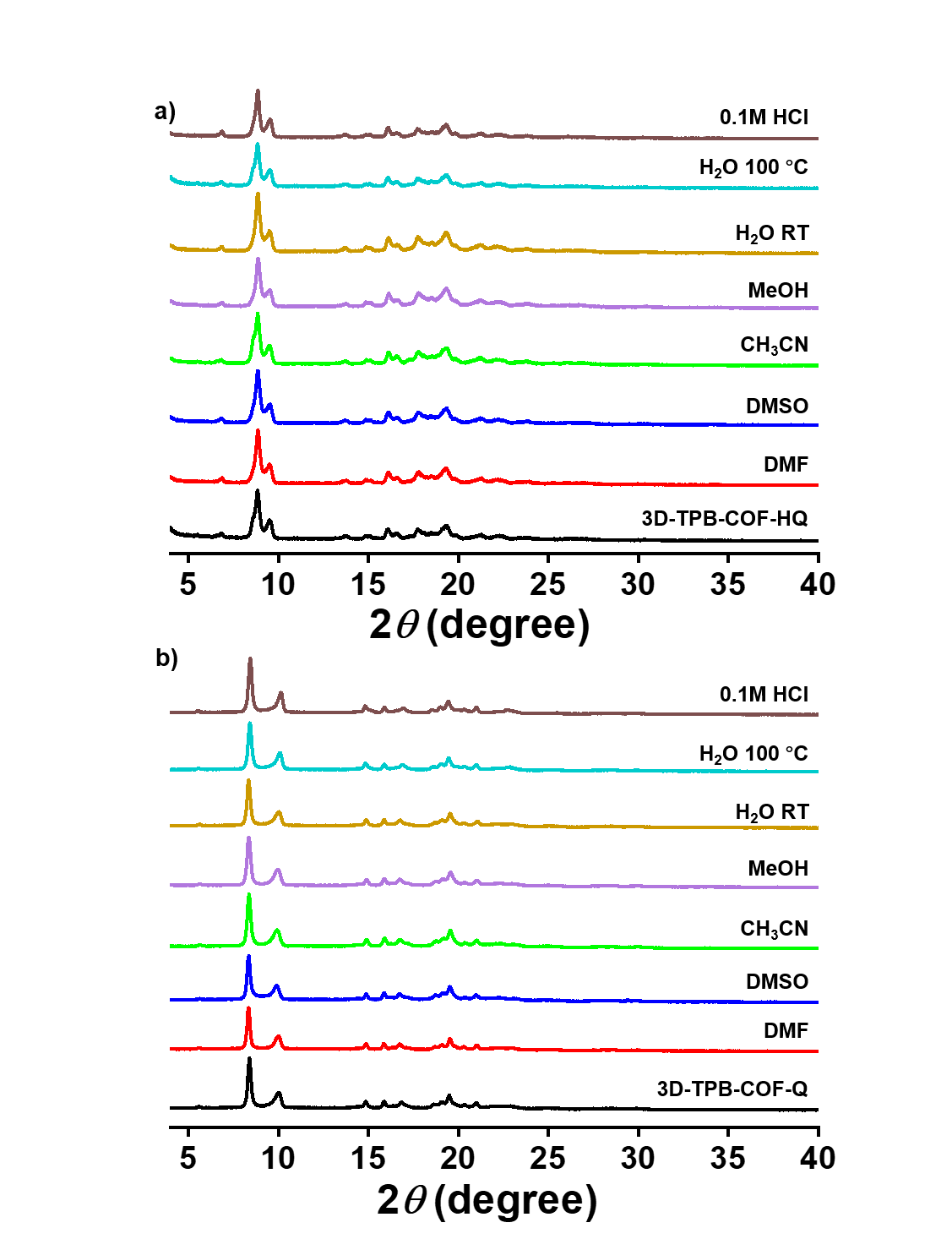


**Supplementary Figure 9∣**PXRD patterns of (a) 3D-TPB-COF-HQ and (b) 3D-TPB-COF-Q after treatment in different solvents for 24 hours.

Supplementary Method 5

**Crystal structure analysis**

The method of cRED data collection was similar with our previous report^2^. The sample was cooled down to 99K by using Gatan cryo-transfer tomography holder in order to reduce the beam damage. During the data collection, the goniometer was rotated continuously while the selected area ED patterns were captured from the crystal simultaneous by a quad hybrid pixel detector (Timepix), using the software of *instamatic*^3^. All the ED patterns were recorded under the spot size 3 with the exposure time 0.5 s.

Data processing was conducted using the software package XDS^4^. For the data solution, six datasets of 3D-TPB-COF-HQ and eight datasets of 3D-TPB-COF-Q were merged in order to improve the data completeness. The structure solution against the cRED data were conducted by *SHELXT* software package^5^. All the position of non-hydrogen atoms (C, N, O) were located by difference electrostatic potential map with the space group of *C*2/*c*. The 3D reciprocal lattice was reconstructed with one of the typical single dataset by the software REDp^6^. In reconstructed 3D reciprocal lattice, 467 ED patterns of 3D-TPB-COF-HQ were recorded with the total tilt rang of 120.1° and 406 ED patterns of 3D-TPB-COF-Q were recorded with the total tilt rang of 103.4°. Powder X-ray diffraction (PXRD) patterns were obtained on a Rigaku Smartlab X-Ray diffractometer with Cu Kα line (λ = 1.5418 Å) focused radiation at 40 kV and 40 mA. The Rietveld refinement with rigid-body constraints was performed on the experimental PXRD using Topas 4.1^7^.


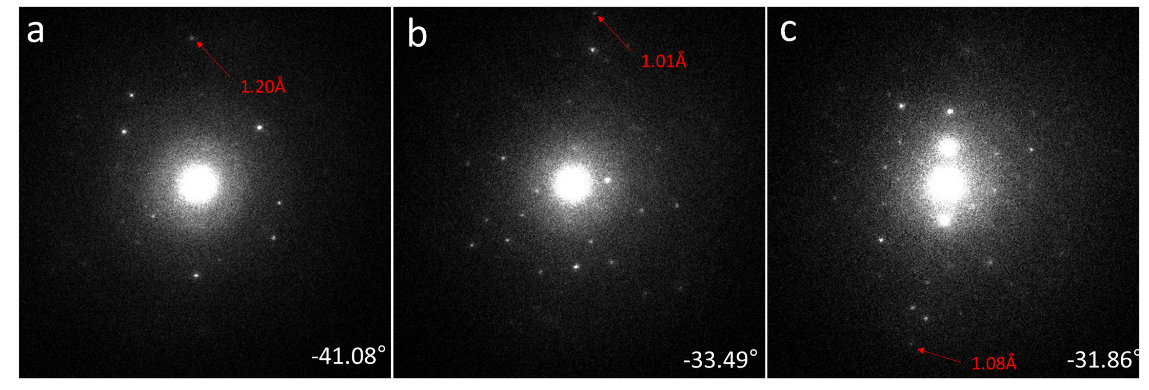


**Supplementary Figure 10∣**Typical SAED patterns of 3D-TPB-COF-HQ under different tilt angle during cRED data collection, with tilt angle of (a) -41.08^o^, (b) -33.49^o^, and (c) -31.86^o^.


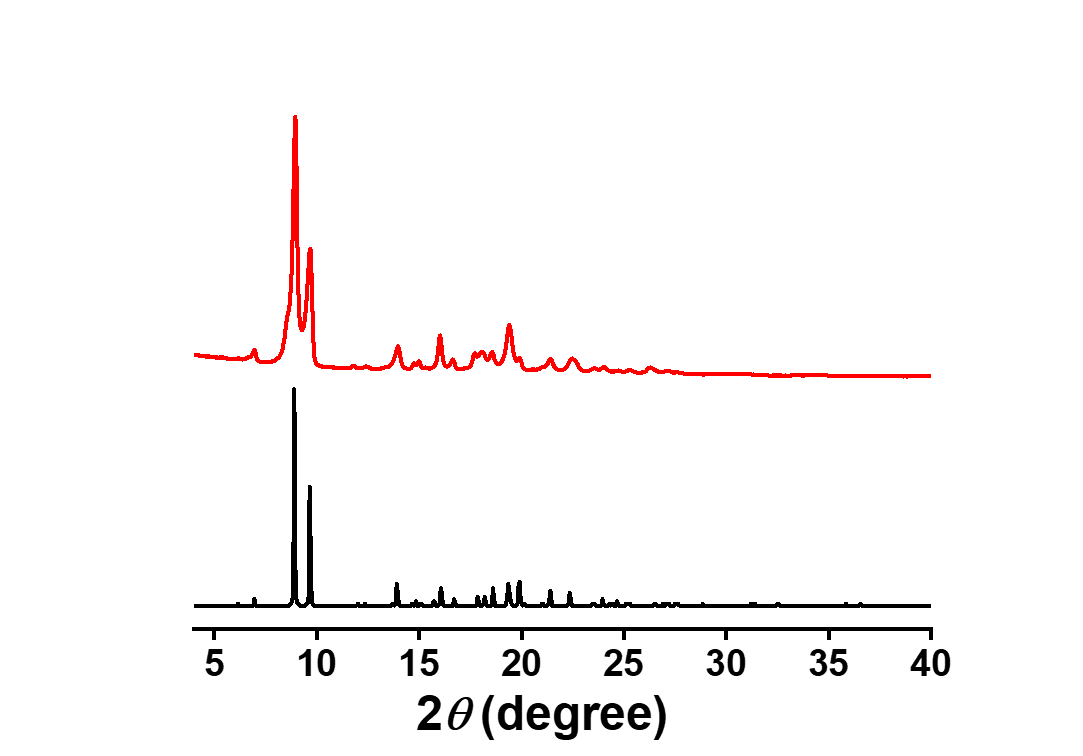


**Supplementary Figure 11∣**PXRD patterns of 3D-TPB-COF-HQ. The experimental patterns are shown in red, the patterns calculated on the basis of the **pts** net in black.

**Supplementary Table 1∣**Fractional atomic coordinates and the unit cell of 3D-TPB-COF-HQ from the Rietveld refinement.

| 3D-TPB-COF-HQ | | Space Group: *C*2/*c*  *a* = 28.761 (90) Å, *b* = 7.645 (1) Å, *c* = 25.535 (30) Å, *α*= *β*= 90°, and *γ* = 94.720 (90)° | | |
| --- | --- | --- | --- | --- |
| C1 | C | 0.0306(0) | 0.0599(5) | 0.2859(5) |
| C2 | C | -0.0285(5) | -0.1877(8) | 0.2821(9) |
| C3 | C | -0.0774(0) | -0.2055(9) | 0.2740(4) |
| C4 | C | -0.0753(0) | -0.4523(8) | 0.3334(9) |
| C5 | C | 0.0617(3) | 0.1731(6) | 0.2629(8) |
| C6 | C | -0.0041(5) | -0.3035(1) | 0.3176(8) |
| C7 | C | -0.1005(8) | -0.3346(0) | 0.2999(8) |
| N1 | N | 0.1086(6) | 0.4333(6) | 0.3792(3) |
| C8 | C | 0.0256(5) | 0.0771(9) | 0.3404(3) |
| C9 | C | 0.0840(0) | 0.3043(6) | 0.3475(5) |
| C10 | C | 0.0530(0) | 0.1949(9) | 0.3708(9) |
| N2 | N | -0.0968(8) | -0.5964(3) | 0.3572(2) |
| C11 | C | -0.0273(9) | -0.4321(3) | 0.3435(3) |
| C12 | C | 0.0889(1) | 0.2905(4) | 0.2934(9) |
| C13 | C | 0.2242(7) | 1.1061(7) | 0.4790(5) |
| C14 | C | 0.2546(9) | 1.1960(1) | 0.4483(8) |
| C15 | C | 0.2792(7) | 1.3418(3) | 0.4684(6) |
| C16 | C | 0.1940(4) | 0.9703(6) | 0.4531(9) |
| C17 | C | -0.1967(1) | -1.0504(9) | 0.4318(1) |
| C18 | C | -0.2235(0) | -0.9671(2) | 0.3907(6) |
| C19 | C | -0.2029(0) | -0.8472(3) | 0.3591(4) |
| C20 | C | -0.1554(6) | -0.8091(4) | 0.3685(9) |
| C21 | C | -0.1279(7) | -0.9005(2) | 0.4076(8) |
| C22 | C | -0.1485(9) | -1.0219(6) | 0.4390(8) |
| C23 | C | 0.1682(1) | 1.0138(0) | 0.4061(0) |
| C24 | C | 0.1441(3) | 0.8861(0) | 0.3766(7) |
| C25 | C | 0.1444(1) | 0.7135(4) | 0.3944(7) |
| C26 | C | 0.1677(9) | 0.6701(9) | 0.4431(6) |
| C27 | C | 0.1927(5) | 0.7983(9) | 0.4723(5) |
| H1 | H | -0.2622(7) | -0.9979(0) | 0.3833(6) |
| H2 | H | -0.2246(7) | -0.7802(3) | 0.3256(0) |
| C28 | C | -0.1360(2) | -0.6665(0) | 0.3396(6) |
| H3 | H | -0.0888(4) | -0.8750(0) | 0.4135(9) |
| H4 | H | -0.1265(9) | -1.0979(5) | 0.4705(4) |
| H5 | H | 0.1669(5) | 1.1551(4) | 0.3918(3) |
| H6 | H | 0.1240(1) | 0.9217(5) | 0.3378(0) |
| C29 | C | 0.1209(5) | 0.5821(6) | 0.3606(3) |
| H7 | H | 0.1663(2) | 0.5306(5) | 0.4586(8) |
| H8 | H | 0.2121(0) | 0.7637(3) | 0.5117(4) |
| O1 | O | 0.2610(1) | 1.1396(2) | 0.3983(7) |
| H9 | H | -0.0982(7) | -0.1135(7) | 0.2459(3) |
| H10 | H | 0.0648(3) | 0.1690(7) | 0.2187(8) |
| H11 | H | 0.0353(3) | -0.2921(2) | 0.3254(9) |
| H12 | H | -0.1402(0) | -0.3449(4) | 0.2940(7) |
| H13 | H | -0.0010(0) | -0.0060(5) | 0.3598(1) |
| H14 | H | 0.0500(6) | 0.2021(3) | 0.4150(9) |
| H15 | H | -0.0071(5) | -0.5213(2) | 0.3730(5) |
| H16 | H | 0.1153(6) | 0.3762(6) | 0.2746(6) |
| H17 | H | -0.1550(9) | -0.6164(3) | 0.3017(0) |
| H18 | H | 0.1134(3) | 0.6121(4) | 0.3170(2) |
| H19 | H | 0.2988(2) | 1.1386(7) | 0.3925(1) |
| C30 | C | 0.000000 | -0.0630(5) | 0.250000 |

**Supplementary Table 2∣**Summary of the Rietveld refinement results of 3D-TPB-COF-HQ

| Name | 3D-TPB-COF-HQ |
| --- | --- |
| Chemical formula | C30H19ON2 |
| Formula Weight/ g mol^-1^ | 423.48 |
| Density/g cm^-3^ | 0.9911(17) |
| Crystal system | Monoclinic |
| Unit cell | *a* = 28.761 (90) Å, *b* = 7.645 (1) Å, *c* = 25.535 (30) Å, *α*= *β*= 90°, and *γ* = 94.720 (90)° |
| Space group | *C*2/*c* |
| Volume/Å^3^ | 5595.77 |
| Z | 8 |
| X-ray source | Cukα |
| Wavelength /Å | 1.5418 |
| 2*θ* | 4-40° |
| Number of reflections | 266 |
| Number of data points | 3601 |
| Refinement method | Rietveld refinement |
| *R_p_* | 3.401% |
| *R_wp_* | 4.739% |
| *R_exp_* | 1.838% |
| GoF | 2.65 |


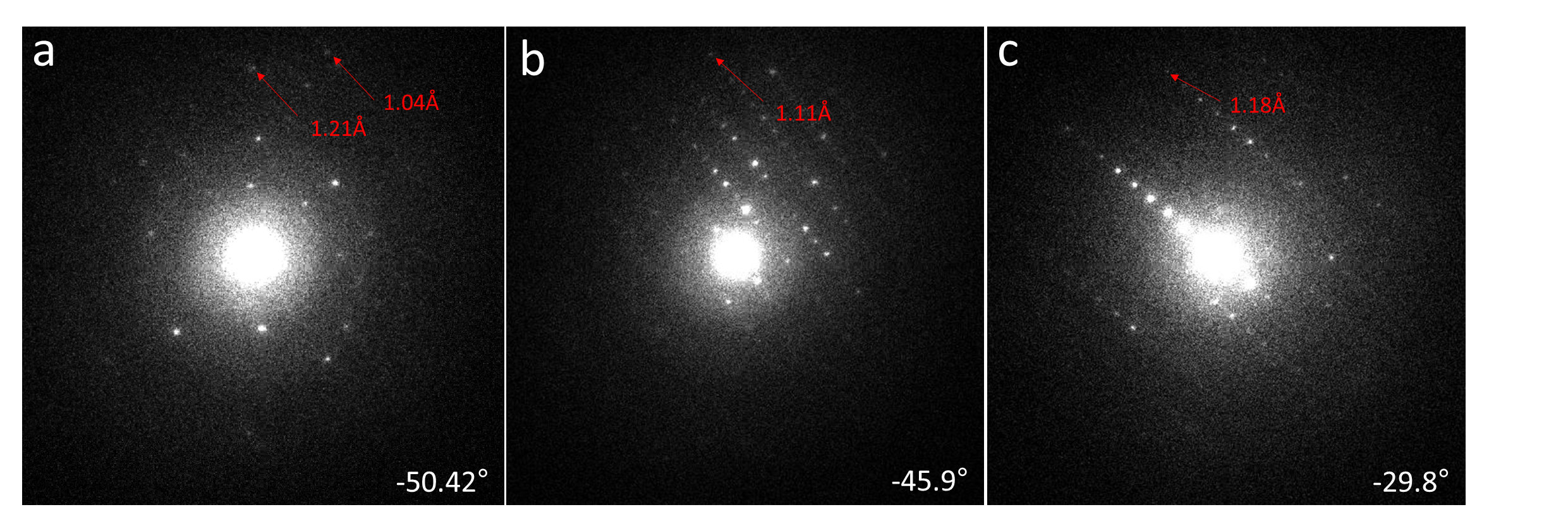


**Supplementary Figure 12∣**Typical SAED patterns of 3D-TPB-COF-Q under different tilt angle during cRED data collection, with tilt angle of (a) -50.42^o^, (b) -45.9^o^, and (c) -29.8^o^.


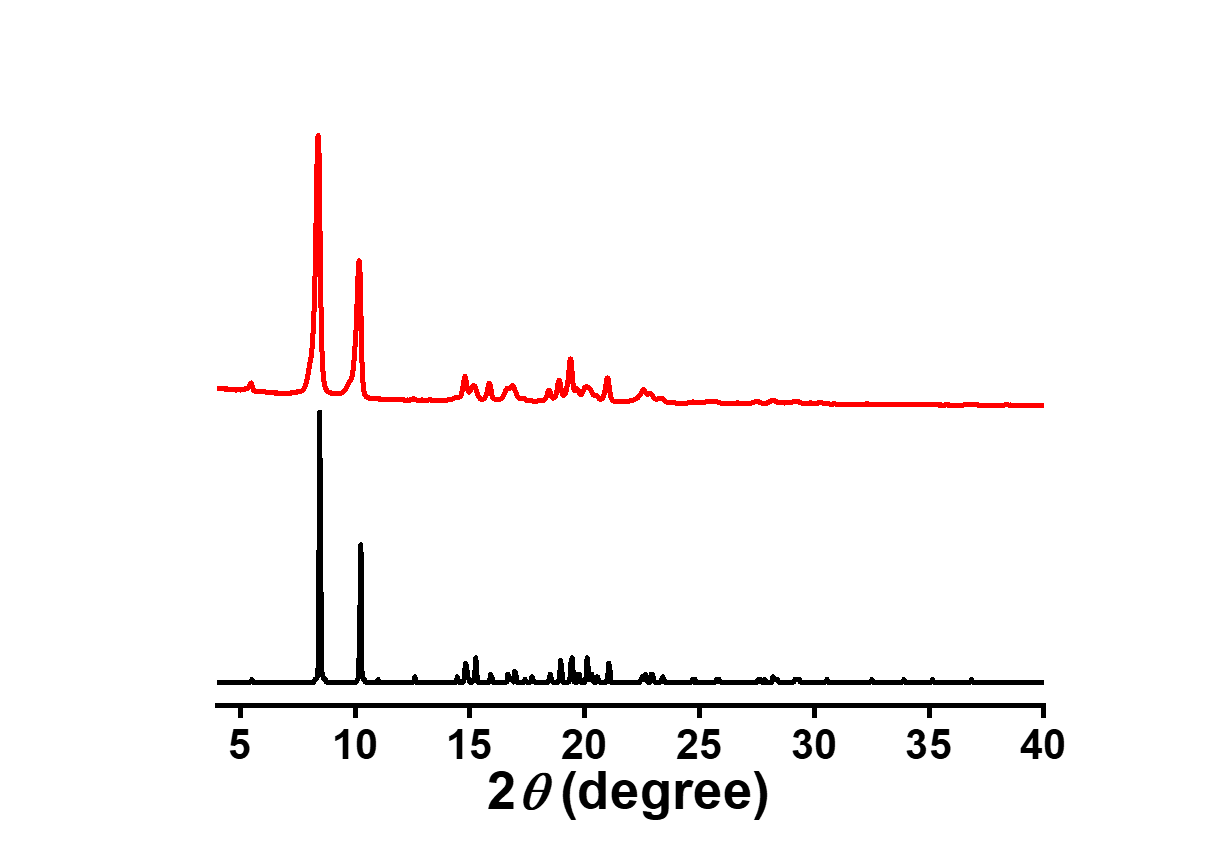


**Supplementary Figure 13∣**PXRD patterns of 3D-TPB-COF-Q. The experimental patterns are shown in red, the patterns calculated on the basis of the **pts** net in black.

**Supplementary Table 3∣**Fractional atomic coordinates and the unit cell of 3D-TPB-COF-Q from the Rietveld refinement.

| 3D-TPB-COF-Q | | Space Group: *C*2/*c*  *a* = 32.797 (30) Å, *b* = 7.199 (10) Å, *c* = 23.699 (30) Å, *α*= *β*= 90°, and *γ* = 101.37 (1)° | | |
| --- | --- | --- | --- | --- |
| H1 | H | 0.6053(0) | 0.0527(7) | 0.3185(0) |
| H2 | H | 0.3704(8) | 0.7751(9) | 0.3069(6) |
| H3 | H | 0.5961(5) | 0.8071(3) | 0.2673(6) |
| H4 | H | 0.5018(4) | 0.8267(8) | 0.3780(8) |
| H5 | H | 0.5718(9) | 0.5894(0) | 0.4265(0) |
| H6 | H | 0.5232(7) | 0.3690(0) | 0.3733(8) |
| H7 | H | 0.3837(5) | 0.0893(9) | 0.3168(6) |
| H8 | H | 0.5350(2) | 0.0503(8) | 0.3233(1) |
| H9 | H | 0.5464(8) | 0.5964(5) | 1.2135(2) |
| H10 | H | 0.4145(0) | 0.3000(0) | 0.2607(0) |
| H11 | H | 0.7126(0) | 0.2439(6) | 0.5215(3) |
| H12 | H | 0.6675(4) | 0.9964(0) | 0.4718(1) |
| H13 | H | 0.6351(7) | 0.3499(6) | 0.3224(7) |
| H14 | H | 0.6785(7) | 0.5956(6) | 0.3722(3) |
| H15 | H | 0.3655(9) | 0.3613(2) | 0.5029(9) |
| H16 | H | 0.4110(3) | 0.5346(7) | 0.4505(2) |
| H17 | H | 0.3058(8) | 0.6323(7) | 0.3120(4) |
| H18 | H | 0.2608(1) | 0.4667(5) | 0.3655(4) |
| C2 | C | 0.6206(0) | 0.0155(4) | 0.3615(8) |
| C3 | C | 0.3851(8) | 0.7253(5) | 0.3491(7) |
| C4 | C | 0.6954(2) | 0.2674(5) | 0.4782(2) |
| C5 | C | 0.6699(5) | 0.1267(3) | 0.4499(3) |
| C6 | C | 0.6478(6) | 0.1559(6) | 0.3936(0) |
| C7 | C | 0.6516(9) | 0.3250(1) | 0.3659(8) |
| C8 | C | 0.6765(2) | 0.4650(9) | 0.3942(5) |
| C9 | C | 0.3527(9) | 0.4194(9) | 0.4611(5) |
| C10 | C | 0.3784(7) | 0.5171(2) | 0.4312(3) |
| C11 | C | 0.3614(5) | 0.5979(8) | 0.3778(6) |
| C12 | C | 0.3194(1) | 0.5714(4) | 0.3533(1) |
| C13 | C | 0.2937(7) | 0.4772(5) | 0.3835(9) |
| C14 | C | 0.3101(0) | 0.4064(7) | 0.4383(2) |
| C15 | C | 0.6983(9) | 0.4380(7) | 0.4505(0) |
| C16 | C | 0.7812(6) | 0.8290(9) | 0.4721(4) |
| C17 | C | 0.7567(5) | 0.6651(6) | 0.4490(1) |
| C18 | C | 0.7245(9) | 0.5913(1) | 0.4785(1) |
| C19 | C | 0.5799(8) | 0.7148(4) | 0.2910(0) |
| C20 | C | 0.4833(4) | 0.9333(8) | 0.3530(7) |
| C21 | C | 0.5671(1) | 0.5879(5) | 0.3800(2) |
| C22 | C | 0.5870(4) | 0.7185(5) | 0.3512(8) |
| C23 | C | 0.5392(5) | 0.4603(0) | 0.3489(2) |
| C24 | C | 0.4169(2) | 0.0796(3) | 0.3185(3) |
| C25 | C | 0.5020(2) | 0.0601(9) | 0.3215(8) |
| C26 | C | 0.5517(9) | 0.5917(3) | 0.2600(4) |
| C27 | C | 0.4405(0) | 0.9380(1) | 0.3497(2) |
| C28 | C | 0.4352(8) | 0.2044(3) | 0.2867(3) |
| C29 | C | 0.4784(0) | 0.1980(8) | 0.2873(2) |
| C30 | C | 0.5315(6) | 0.4578(6) | 0.2879(3) |
| N1 | N | 0.4206(8) | 0.7922(1) | 0.3750(6) |
| N2 | N | 0.6134(1) | 0.8566(1) | 0.3836(2) |
| O1 | O | 0.7640(5) | 0.5866(9) | 0.4064(0) |
| C1 | C | 0.500000 | 0.3272(5) | 0.250000 |

**Supplementary Table 4∣**Summary of the Rietveld refinement results of 3D-TPB-COF-Q

| Name | 3D-TPB-COF-Q |
| --- | --- |
| Chemical formula | C30H18ON2 |
| Formula Weight/ g mol^-1^ | 422.48 |
| Density/g cm^-3^ | 1.008(54) |
| Crystal system | Monoclinic |
| Unit cell | *a* = 32.797 (30) Å, *b* = 7.199 (10) Å, *c* = 23.699 (30) Å, *α*= *β*= 90°, and *γ* = 101.37 (1)° |
| Space group | *C*2/*c* |
| Volume/Å^3^ | 5485.85 |
| Z | 8 |
| X-ray source | Cukα |
| Wavelength /Å | 1.5418 |
| 2*θ* | 4-40° |
| Number of reflections | 266 |
| Number of data points | 3601 |
| Refinement method | Rietveld refinement |
| *R_p_* | 4.500% |
| *R_wp_* | 6.827% |
| *R_exp_* | 2.412% |
| GoF | 2.91 |

Supplementary Method 6

**Redox-responsive study of TPB-HQ**

**Supplementary Figure 14∣**Redox switching between TPB-HQ and TPB-Q.

**Oxidation reaction:** We choose *p*-benzoquinone as the oxidant to oxidize TPB-HQ. The reaction was performed as follow: TPB-HQ (50.0 mg, 0.1 mmol) and BQ (86.3 mg, 0.8 mmol) were added to a flask containing acetonitrile (20 mL). The mixture was heated for 23 h at 60 °C. After cooling to room temperature, the solvent was removed under reduced pressure. Then the residues were collected for ^1^H NMR study. ^1^H NMR spectroscopy confirm the quantitative conversion of TPB-HQ and the formation of TPB-Q, the additional peaks belong to BQ and quinol.


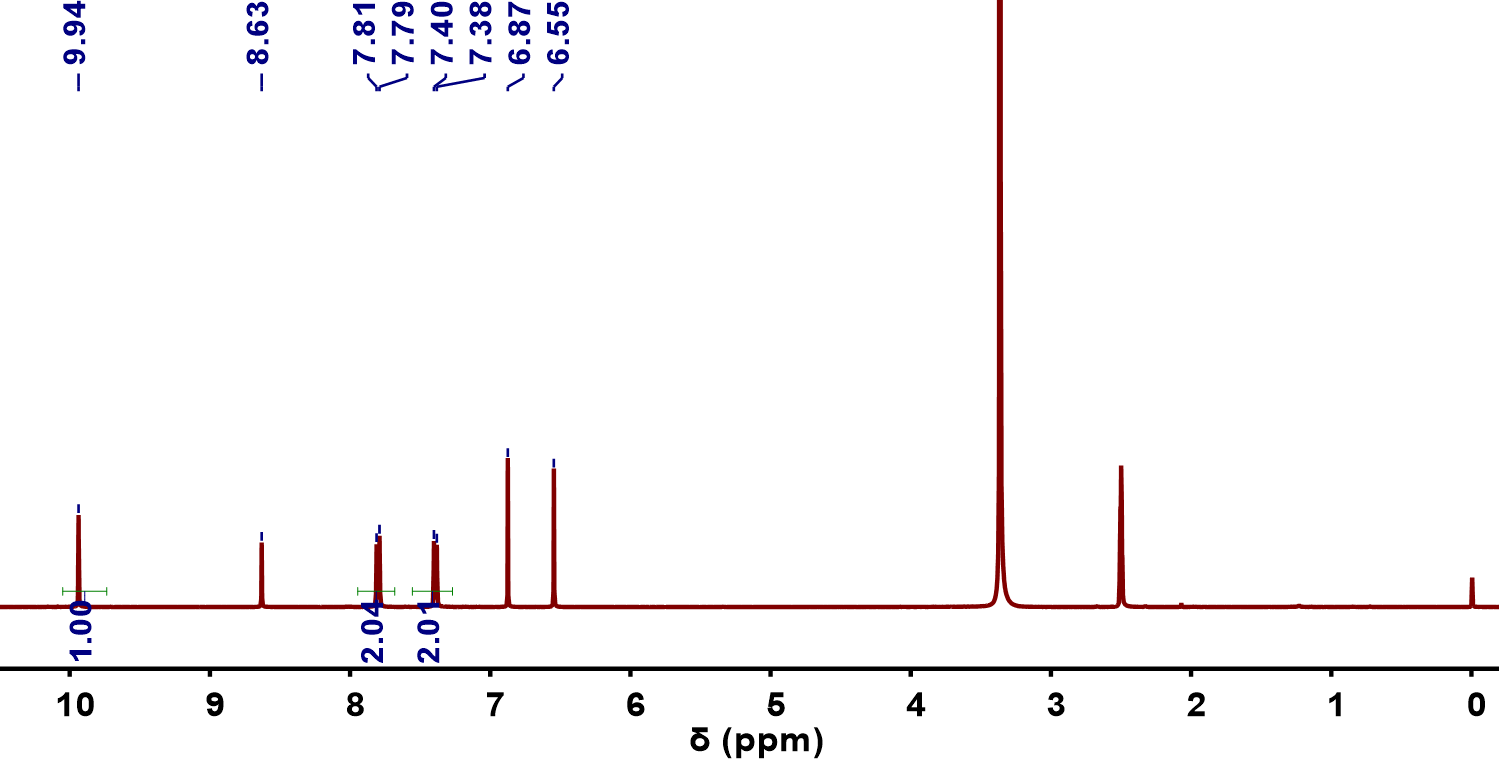


**Supplementary Figure 15∣**^1^H NMR (400 MHz, DMSO-*d*_6_) spectrum of TPB-HQ oxidation. *Note*: the peak at 6.55 ppm belongs to *p*-benzoquinone, whereas the peaks at 6.87 and 8.63 ppm belong to hydroquinone.

**Reduction reaction:** we choose ascorbic acid as the reductant to reduce TPB-Q. The reaction was performed as follow: TPB-Q (20 mg, 0.04 mmol) was added to a flask containing a solution of ascorbic acid in DMF (5 mL, 30 mmol L^-1^), which was stirred for 1 h at 30 °C. Subsequently, deionized water (20 mL) was added and precipitate appeared. The precipitate was collected and washed with a large amount of water (50 mL), dried in vacuum (17.0 mg, 85%). ^1^H NMR spectroscopy confirm the complete formation of TPB-HQ.

**Supplementary Figure 16∣**^1^H NMR (400 MHz, DMSO-*d*_6_) spectrum of TPB-HQ formed by reduction of TPB-Q.

Supplementary Method 7

**Redox-triggered transformation in 3D-TPB-COF-HQ**

**Supplementary Figure 17∣**Reversible redox-triggered transformation between 3D-TPB-COF-HQ and 3D-TPB-COF-Q.

**Oxidation of 3D-TPB-COF-HQ:** A Pyrex tube was charged with 3D-TPB-COF-HQ (20.0 mg), *p*-benzoquinone (21.8 mg) and CH_3_CN (5.0 mL). The tube was sealed under air and stirred at 60 °C for different time. The resulting precipitate was filtered and exhaustively washed by CH_3_CN, dried under vacuum. The degree of oxidation was determined by ^1^H NMR of digested powders, and about 90% of hydroquinone in COF skeleton was oxidized to quinone within 90 min. After that, there is no obvious improvement for the degree of oxidation.

**Reduction reaction for 3D-TPB-COF-Q:** 3D-TPB-COF-Q (20 mg) was added to a tube containing a solution of ascorbic acid in MeOH (5.0 mL, 0.2 mol L^-1^), and the mixture was stirred at 30 °C for different time. The resulting precipitate was filtered and exhaustively washed by MeOH and DCM, dried under vacuum. The degree of reduction was determined by ^1^H NMR of digested powders, and all of quinone units in COF skeleton was reduced to hydroquinone within 90 min.

**Supplementary Figure 18∣**FT-IR spectra during the redox process of 3D-TPB-COF-HQ. (a) 3D-TPB-COF-HQ; (b) oxidation for 2 min; (c) oxidation for 40 min; (d) 3D-TPB-COF-Q (oxidation for 90 min); (e) reduction for 30 min; (f) reduction for 60 min; (g) 3D-TPB-COF-HQ(R) (reduction for 90 min). *Note:* the peaks appeared at 1650 cm^−1^ should be assigned to the stretch vibrational frequency of C=O groups in quinone units.


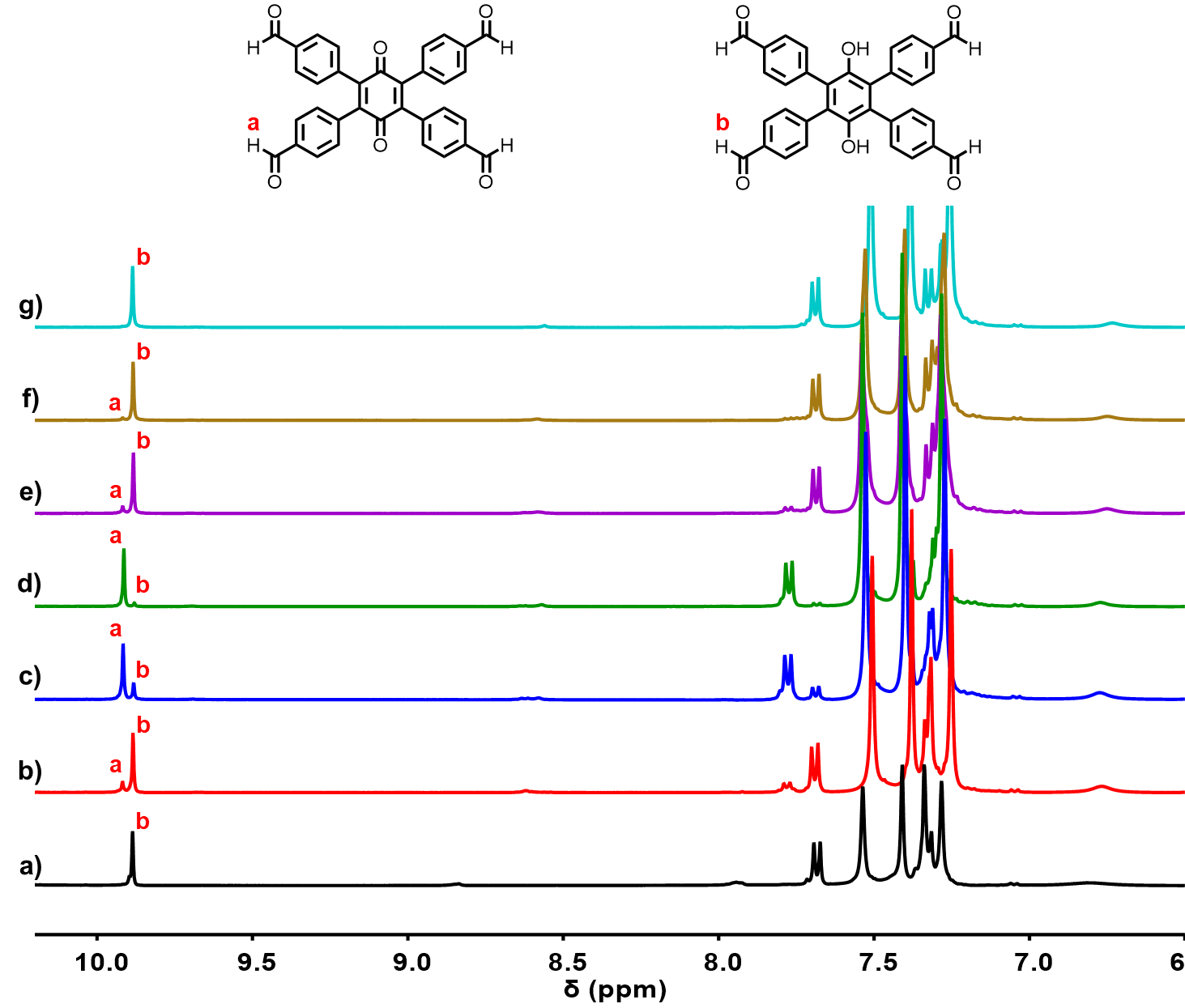


**Supplementary Figure 19∣**^1^H NMR spectra of digested powders. (a) 3D-TPB-COF-HQ; (b) oxidation for 2 mins; (c) oxidation for 40 mins; (d) 3D-TPB-COF-Q (oxidation for 90 mins); (e) reduction for 30 mins; (f) reduction for 60 mins; (g) 3D-TPB-COF-HQ(R) (reduction for 90 mins).

**Further oxidation and reduction cycles:** We further studied the reversible transformation of 3D-TPB-COF-HQ via oxidation/reduction. Under the same redox condition, such reversible transformation is repeatable without losing of the crystallinity.


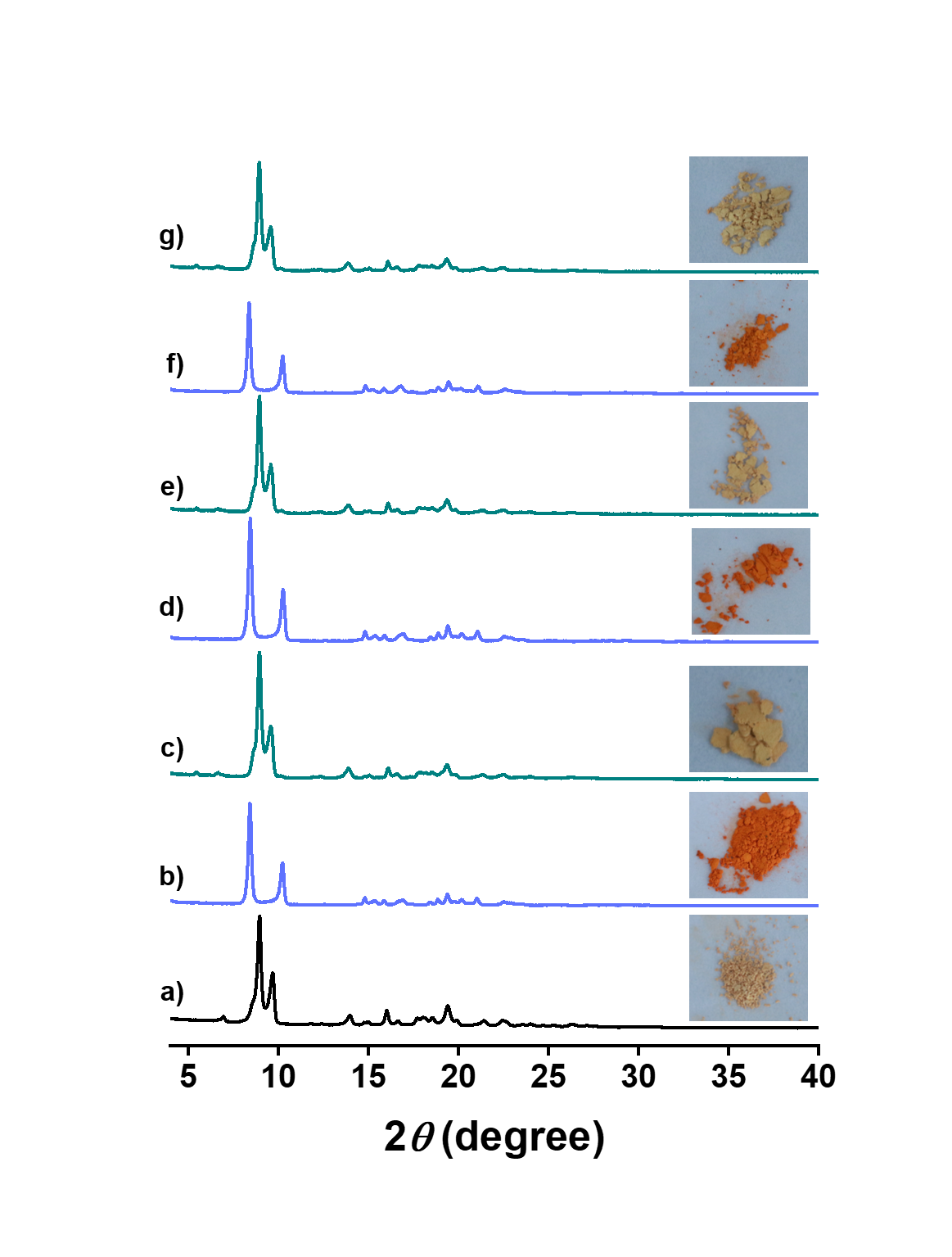


**Supplementary Figure 20∣**PXRD patterns of 3D COFs during the reversible transformation in three oxidation/reduction cycles. (a) 3D-TPB-COF-HQ; (b) 1^st^ oxidation; (c) 1^st^ reduction; (d) 2^nd^ oxidation; (e) 2^nd^ reduction; (f) 3^rd^ oxidation; (g) 3^rd^ reduction. Inset: the pictures of corresponding 3D COFs.


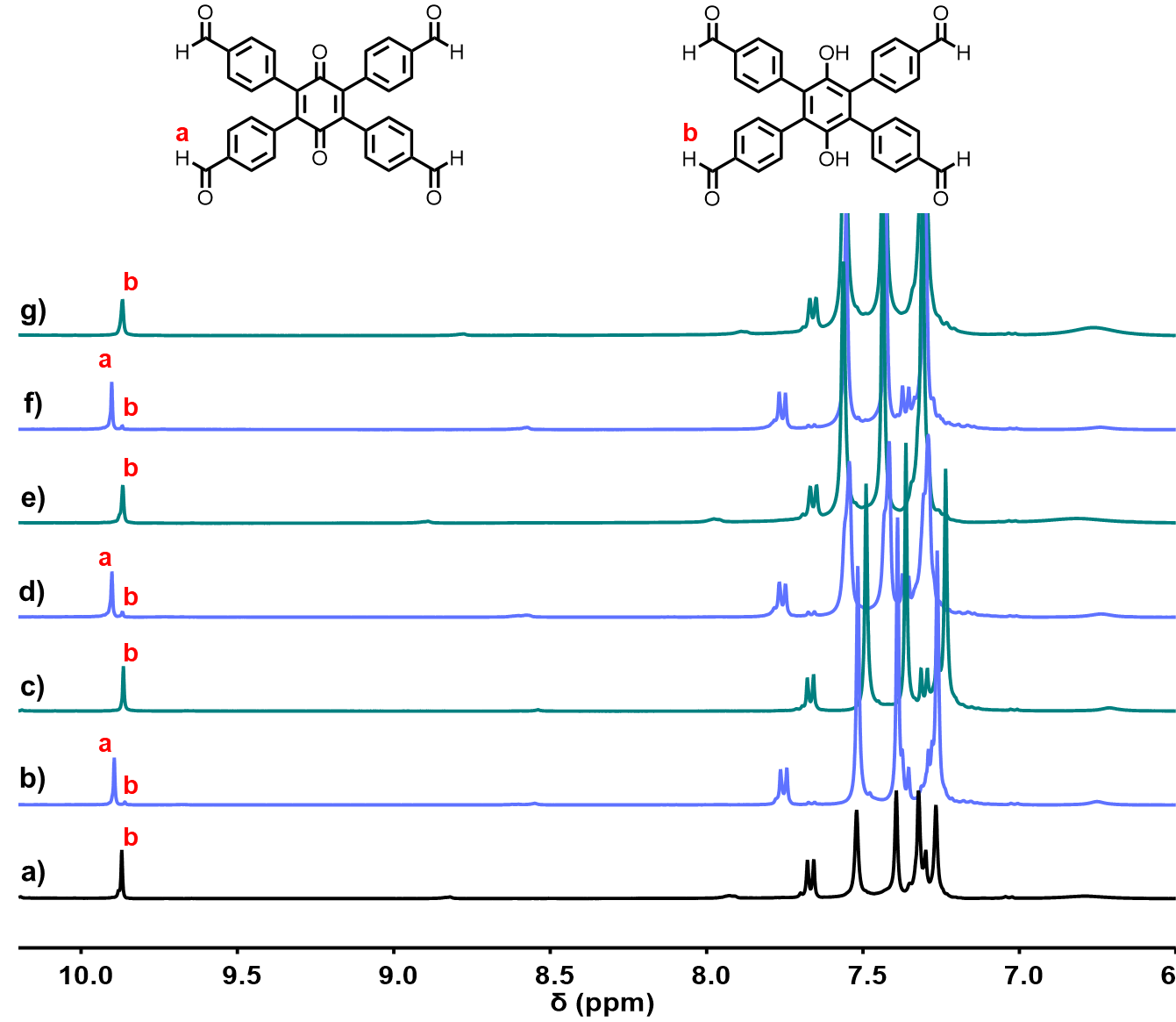


**Supplementary Figure 21∣**^1^H NMR spectra of digested powders during the reversible transformation in three oxidation/reduction cycles. (a) 3D-TPB-COF-HQ; (b) 1^st^ oxidation; (c) 1^st^ reduction; (d) 2^nd^ oxidation; (e) 2^nd^ reduction; (f) 3^rd^ oxidation; (g) 3^rd^ reduction.


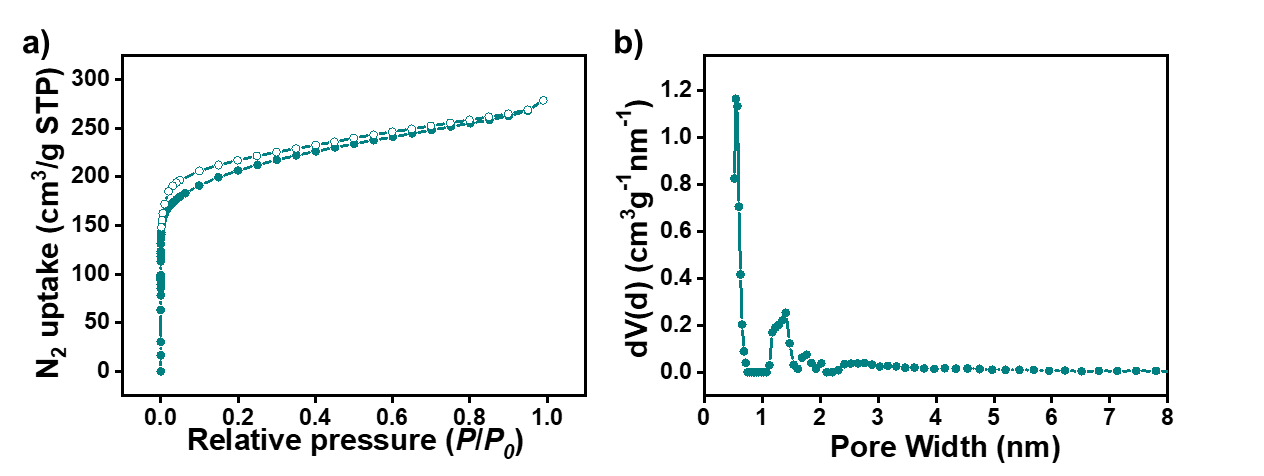


**Supplementary Figure 22∣**N_2_ adsorption−desorption isotherm of 3D-TPB-COF-HQ(R) after three cycles at 77 K (a) and its pore size distribution (b).

Supplementary Method 8

**Gas absorption and separation**

In order to compare the efficacy of these 3D COFs for CO_2_/N_2_ separation, the Ideal Adsorbed Solution Theory (IAST) of Myers and Prausnitz along with pure component isotherm fits was used to determine the molar loadings in the mixture for specified partial pressures in the bulk gas phase ^8,9^.

The pure component loadings of 3D COFs at 273 K were fitted with a single-site Langmuir-Freundlich model (Eq. 1), where *a* is saturation capacity and *b* and *c* are constants.

$N=\frac{{abP}^{c}}{{1+bP}^{c}}$ (Eq. 1)

The adsorption selectivity, *S_ads_*, is for binary mixtures defined by Eq. 2, where *x_i_* is the mole fractions of component i in the adsorbed phases and *y_i_* is the mole fractions of component i in the bulk phases.

$S_{ads}=\frac{x_{1}/x_{2}}{y_{1}/y_{2}}$ (Eq. 2)

To determine the binding affinity of 3D-TPB-COF-HQ, 3D-TPB-COF-Q and 3D-TPB-COF-HQ(R) for CO_2_, we estimated the *Q_st_* using the virial method based on two independent temperature gas adsorption isotherms (273 K and 298K). In each case, the data were fitted using the following equation 3:

$\ln P= \ln N+ \frac{1}{T}\sum_{i=0}^{m} a_{i}N^{i}+ \sum_{i=0}^{n} b_{i}N^{i}$ (Eq. 3)

Where, *P* is the pressure, mmHg; *N* is the adsorption capacity, mg g^-1^; *T* is the absolute temperature, K; *a_i_* and *b_i_* are virial coefficients, and *m* and *n* are coefficients used to describe the isotherms, usually m≤6 and n≤3.

The following equation (Eq. 4) was used to calculate the isosteric heat of adsorption, where *R* is the universe gas constant.

$Q_{st}= -R\sum_{i=0}^{m} a_{i}N^{i}$ (Eq. 4)


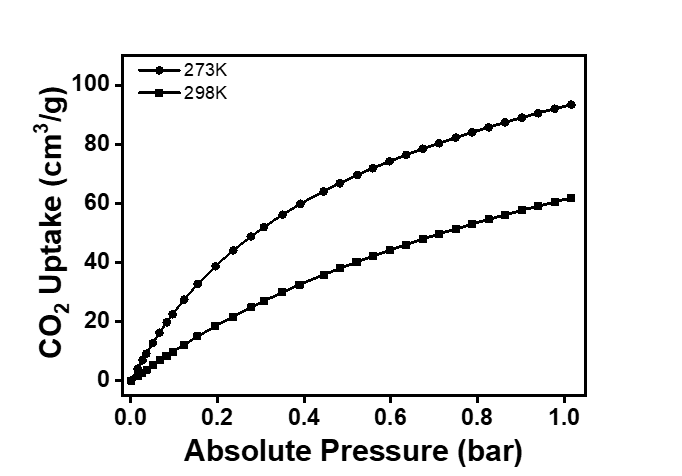


**Supplementary Figure 23∣**CO_2_ adsorption of 3D-TPB-COF-HQ at 273 K (with circle symbol) and 298 K (with square symbol).


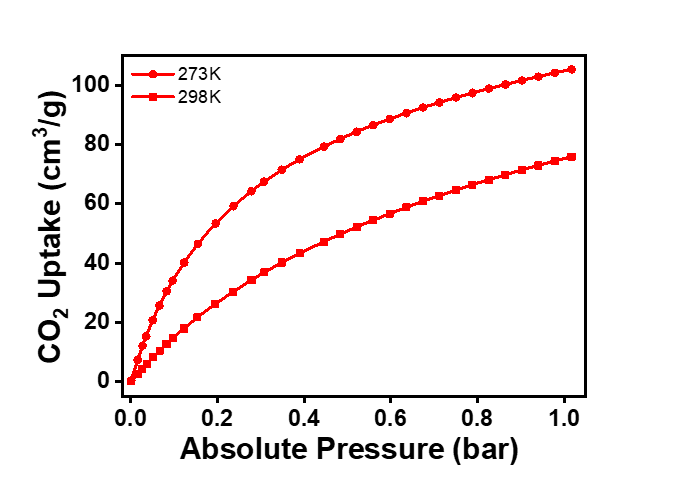


**Supplementary Figure 24∣**CO_2_ adsorption of 3D-TPB-COF-Q at 273 K (with circle symbol) and 298 K (with square symbol).


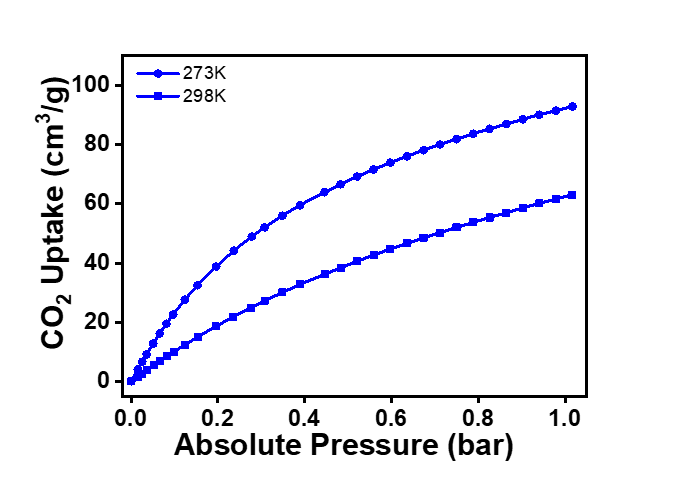


**Supplementary Figure 25**∣CO_2_ adsorption of 3D-TPB-COF-HQ(R) at 273 K (with circle symbol) and 298 K (with square symbol).

**Supplementary Table 5∣**CO_2_ uptake and *Q*_st_ value of 3D COFs.

| 3D COFs | CO_2_ uptake (cm^3^ g^-1^) | | *Q*_st_ (kJ mol^-1^) |
| --- | --- | --- | --- |
|  | 273 K | 298 K |  |
| 3D-TPB-COF-HQ | 93.4 | 62.8 | 23.5 |
| 3D-TPB-COF-Q | 105.4 | 75.9 | 29.0 |
| 3D-TPB-COF-HQ(R) | 92.9 | 63 | 23.7 |

Supplementary Figures

**^1^H and ^13^C NMR Spectra**

**Supplementary Figure 26∣**^1^H NMR spectrum (CDCl_3_, 400 MHz) of compound **1**.

**Supplementary Figure 27∣**^13^C NMR spectrum (CDCl_3_, 400 MHz) of compound **1**.

**Supplementary Figure 28∣**^1^H NMR spectrum (CDCl_3_, 400 MHz) of compound **3**.

**Supplementary Figure 29∣**^1^H NMR spectrum (DMSO-*d*_6_, 400 MHz) of TPB-HQ.

**Supplementary Figure 30∣**^13^C NMR spectrum (DMSO-*d*_6_, 400 MHz) of TPB-HQ.

**Supplementary Figure 31∣**^1^H NMR spectrum (DMSO-*d*_6_, 400 MHz) of TPB-Q.

**Supplementary Figure 32∣**^13^C NMR spectrum (DMSO-*d*_6_, 400 MHz) of TPB-Q.

**Supplementary References**

1. Ganesan, P. et al. Tetrahedral n-type materials: efficient quenching of the excitation of p-type polymers in amorphous films. *J. Am. Chem. Soc.* **127**, 14530−14531 (2005).
2. Gao, C. et al. Isostructural Three-Dimensional Covalent Organic Frameworks. *Angew. Chem. Int. Ed.* **58**, 9770−9775 (2019).
3. Smeets, S., Wang, B., Cichocka, M. O., Angstrom, J. & Wan, W. (2018, April 11) Instamatic (Version 0.6) Zenodo. <https://doi.org/10.5281/zenodo.1217026>
4. Wan, W., Sun, J., Su, J., Hovmöller, S. & Zou, X. Three-dimensional rotation electron diffraction: software RED for automated data collection and data processing. *J Appl Cryst* 46:1863−1873 (2013).
5. Sheldrick, G. M. *SHELXT*-Integrated space-group and crystal-structure determination, [*Acta Cryst*](https://journals.iucr.org/a) [A71](https://journals.iucr.org/a/contents/backissues.html)(1):3−8 (2015).
6. Kabsch, W. Integration, scaling, space-group assignment and post-refinement. *Acta Cryst* D66:125−132 (2010).
7. Coelho, A. A. TOPAS-ACADEMIC v5.0; 2012.
8. Myers, A. L. & Prausnitz, J. M. Thermodynamics of mixed-gas adsorption. *AIChE. J.* **11**, 121−127 (1965).
9. Zhu, Y., Long, H. & Zhang, W. Imine-linked porous polymer frameworks with high small gas (H_2_, CO_2_, CH_4_, C_2_H_2_) uptake and CO_2_/N_2_ selectivity. *Chem. Mater.* **25**, 1630−1635 (2013).
